# Supplementary material for: CD33 rs2455069 SNP: Correlation with Alzheimer’s Disease and Hypothesis of Functional Role
Source: Int J Mol Sci. 2022 Mar 26;23(7):3629. doi: 10.3390/ijms23073629 (PMC8998932; doi:10.3390/ijms23073629)
Supplement: Supplementary file 1 [file ijms-23-03629-s001.zip › Supplementary_Materials.pdf]

Supplementary Materials for

CD33 rs2455069 SNP: correlation with Alzheimer's disease and hypothesis of functional role

Fabiana Tortora [1,†], Antonella Rendina [1,†], Antonella Angiolillo [2], Alfonso Di Costanzo [2], Francesco Aniello [3], Aldo Donizetti [1,3,*], Ferdinando Febbraio [1,‡], Emilia Vitale [1,‡,*]

[1] Institute of Biochemistry and Cell Biology, National Research Council (CNR), Via Pietro Castellino 111, 80131 Naples, Italy
[2] Centre for Research and Training in Medicine of Aging, Department of Medicine and Health Science "V. Tiberio", University of Molise, 86100 Campobasso, Italy
[3] Department of Biology, University of Naples Federico II, 80126 Naples, Italy
* Correspondence to: aldo.donizetti@unina.it, emilia.vitale@cnr.it.

† These authors contributed equally (co-first).

‡ These authors contributed equally (co-last).

Script and configuration file used for *in silico* analysis

<u>Script to automate docking analysis</u>

```
#! /bin/bash

# Reading from step1_pdbreader1.pdbqt to step1_pdbreader100.pdbqt files in the directory
for p in step1_pdbreader*.pdbqt; do

# Extract the filename without .pdbqt
	c=`basename $p .pdbqt`

# Show the filename of processing protein on video
	echo Processing protein $c

# Pass the command to the software autodock vina
	vina --config config.txt --receptor $p --ligand ligand_sialic_acid.pdbqt --out ${c}_out.pdbqt --log ${c}_log.txt
done
```

<u>Vina config file (config.txt) used to define the grid box spatial position</u>

```
center_x = 46.894
center_y = 93.34
center_z = 69.593

size_x = 50
size_y = 60
size_z = 84

exhaustiveness = 16
```

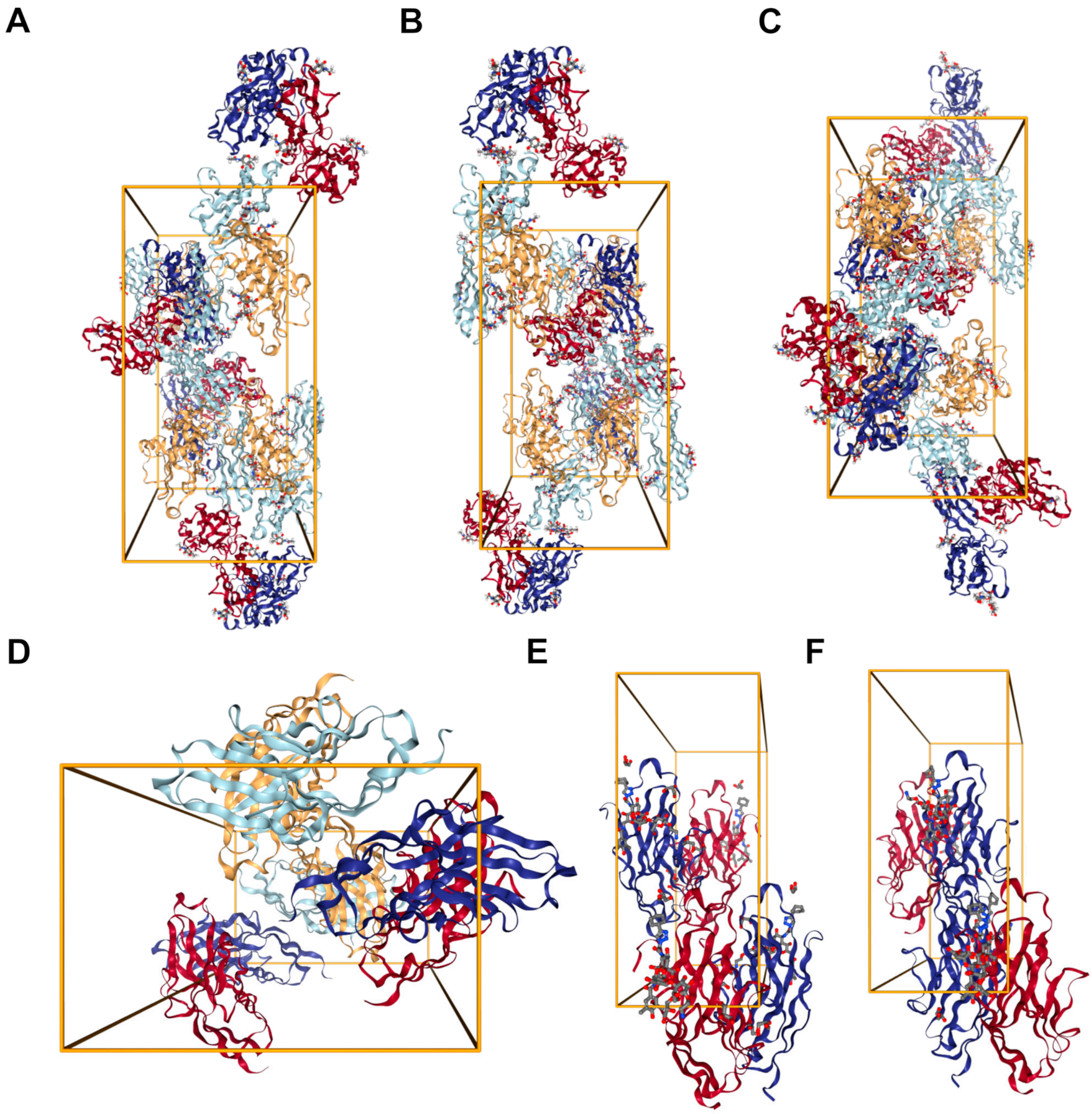

Supplementary Figure S1. Unit cell representation of CD33 structures resolved by X-RAY diffraction. CD33 two domain structures in the crystallographic unit cell of PDB ID (**A**) 5IHB, (**B**) and 5J06 (**C**) 5J0B. CD33 one domain structures in the crystallographic unit cell of PDB ID (**D**) 6D48, (**E**) 6D49 and (**F**) 6D4A

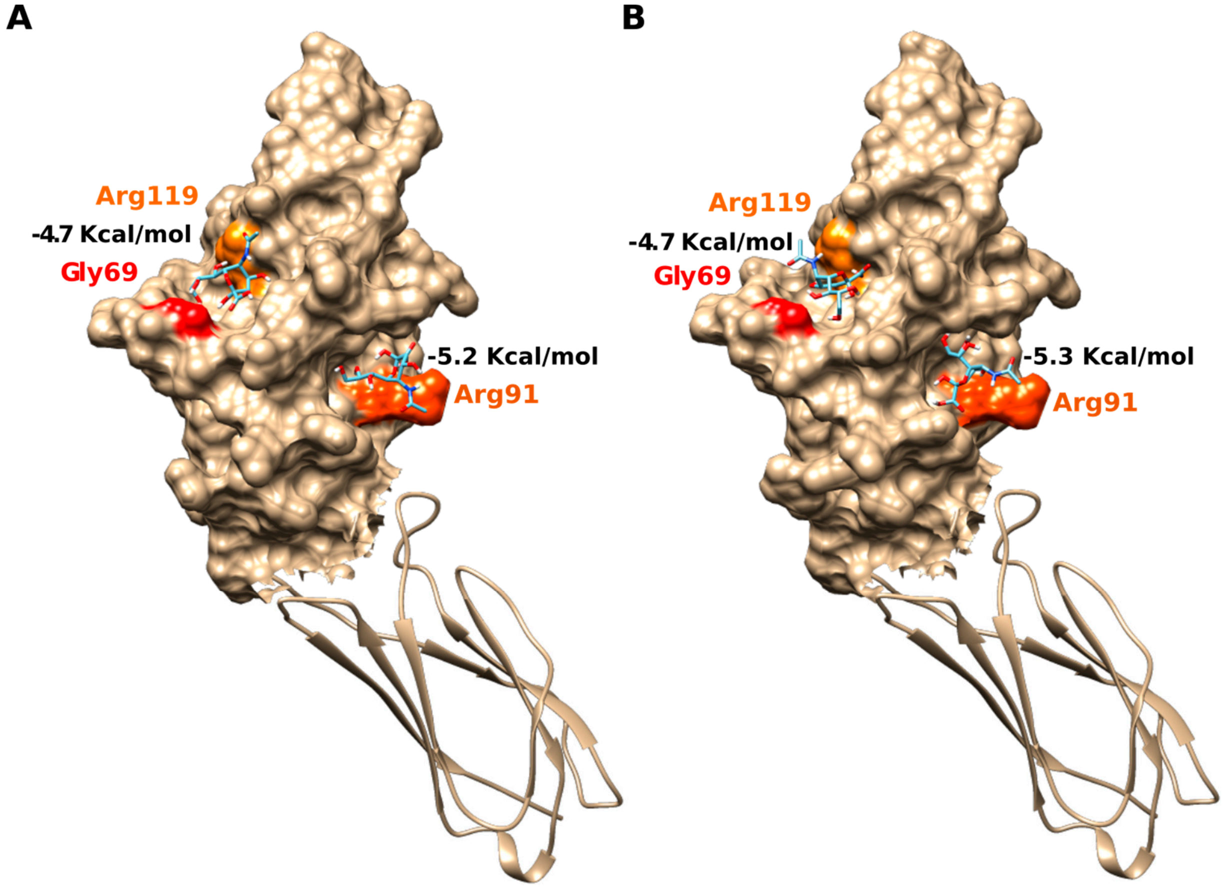

Supplementary Figure S2. Sialic acid interactions with monomers of the CD33. Surface representation of binding domain of the CD33 variant with Gly69 (colored in red) by using PDB ID 5J06 (**A**) or 5J0B (**B**). The areas in orange indicate the binding sites for sialic acid (stick representation) at level of positively charged arginine 91 and 119. Binding energy values in Kcal/mol are reported for each binding site for sialic acid.

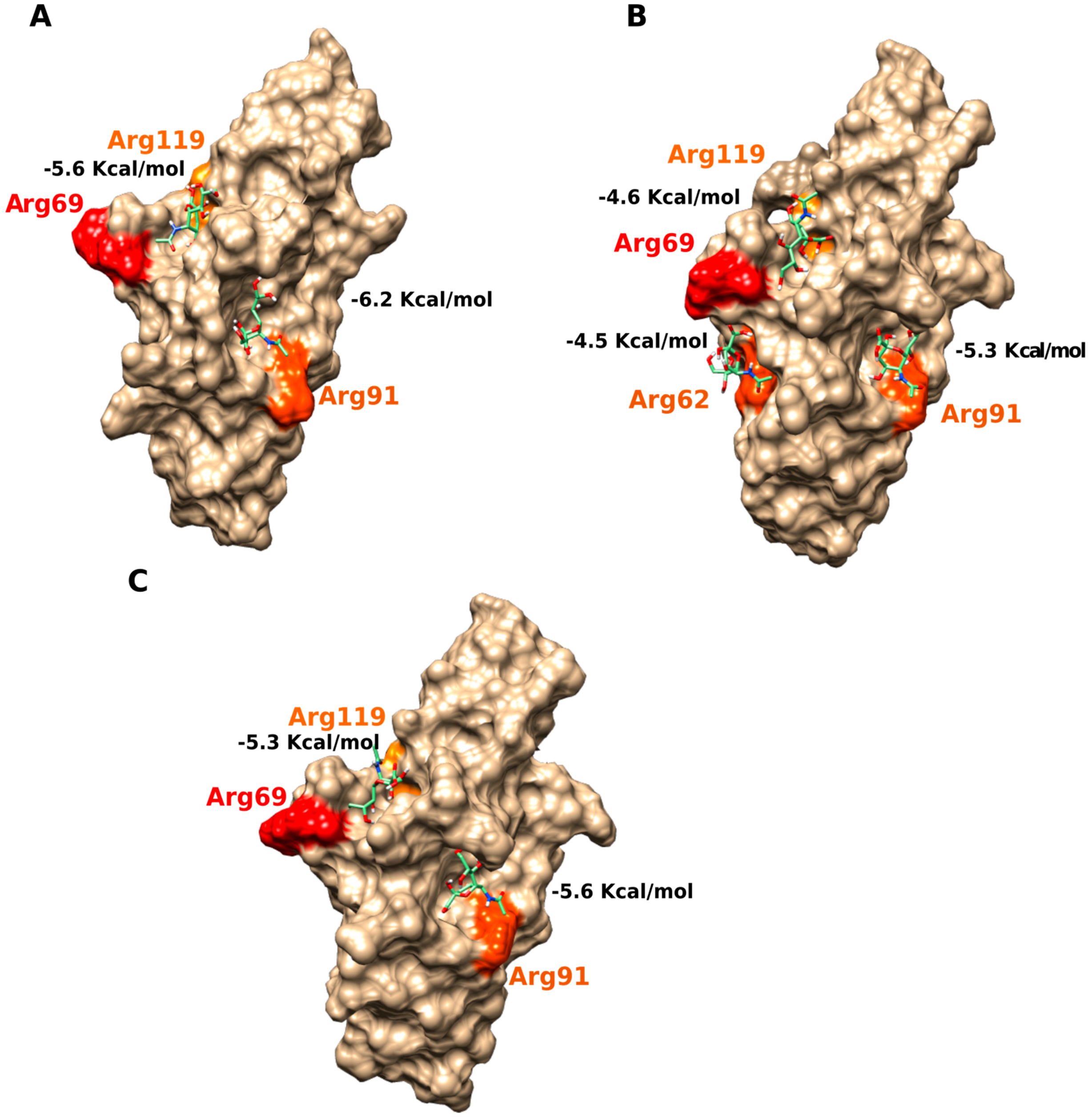

Supplementary Figure S3. Sialic acid interactions with monomers of the CD33. Surface representation of binding domain of the CD33 variant with Arg69 (colored in red) by using PDB ID 6D48 (**A**), 6D49 (**B**) and 6D4A (**C**). The areas in orange indicate the binding sites for sialic acid (stick representation) at level of positively charged arginine 62, 91 and 119. Binding energy values in Kcal/mol are reported for each binding site for sialic acid.

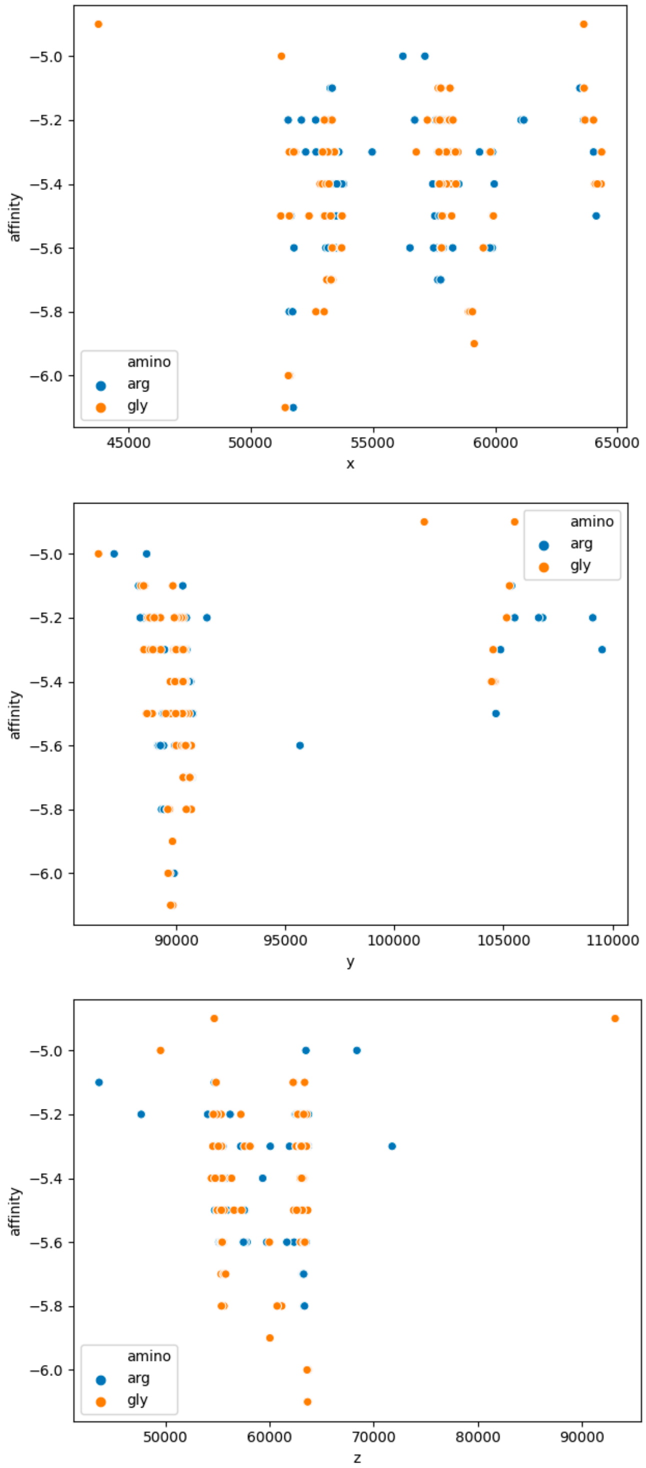

Supplementary Figure S4. Plot of affinity binding vs spatial coordinates of sialic acid molecules by virtual screening analysis. Bidimensional plot of carbon 1 atomic coordinate (x, y and z) of the best 200 ligand-structures against the affinity values (in kcal/mol), obtained by docking analysis, of sialic acid binding the CD33 receptor with Arg (blue) or Gly (orange) residue in position 69.

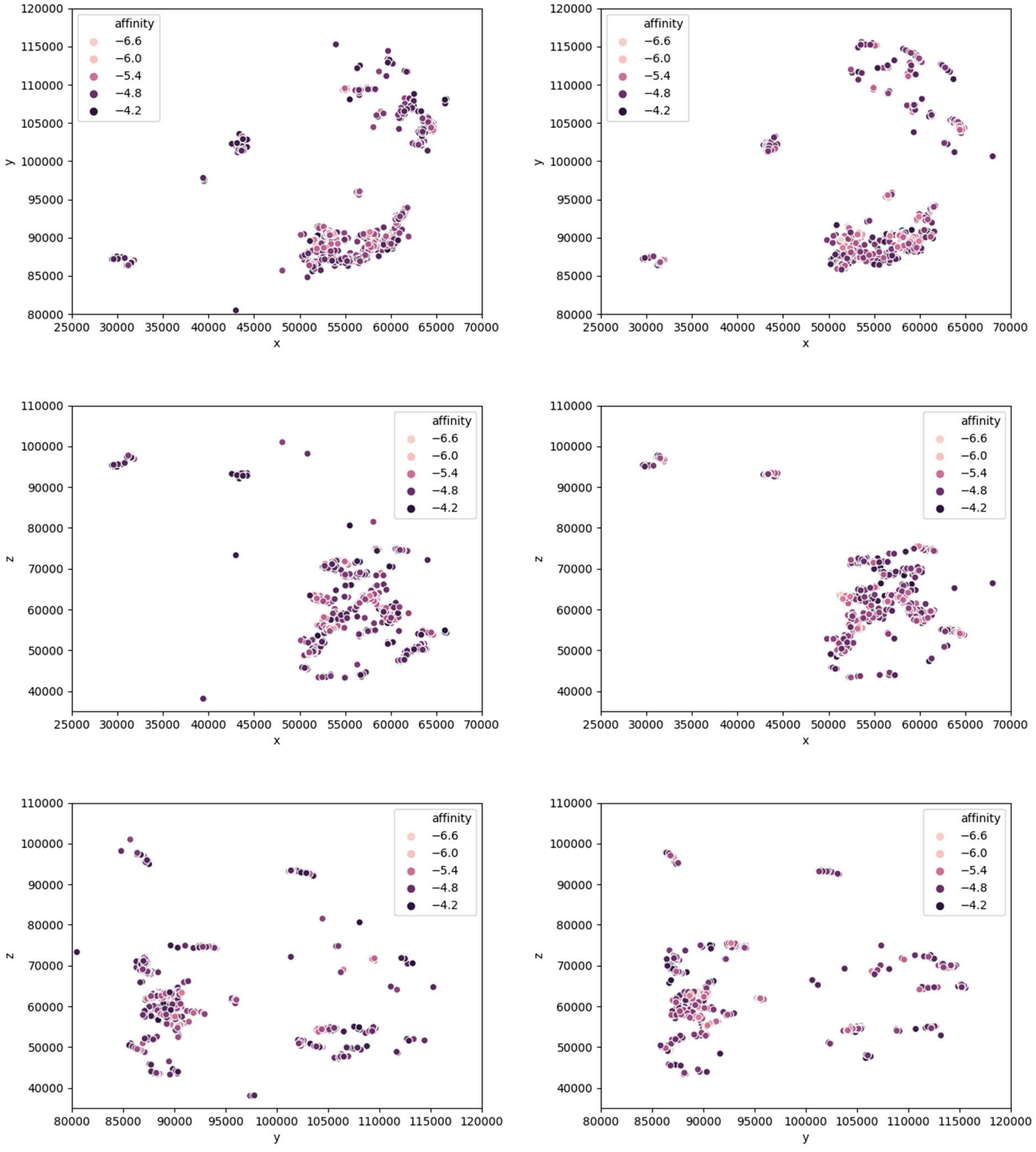

Supplementary Figure S5. Plot of spatial coordinates of sialic acid molecules obtained by docking analysis. Bidimensional plot of atomic coordinate of carbon 1 in the sialic acid structures binding the CD33 receptor (PDB ID 5IHB) with the arginine (left) or glycine (right) residue in position 69.

A

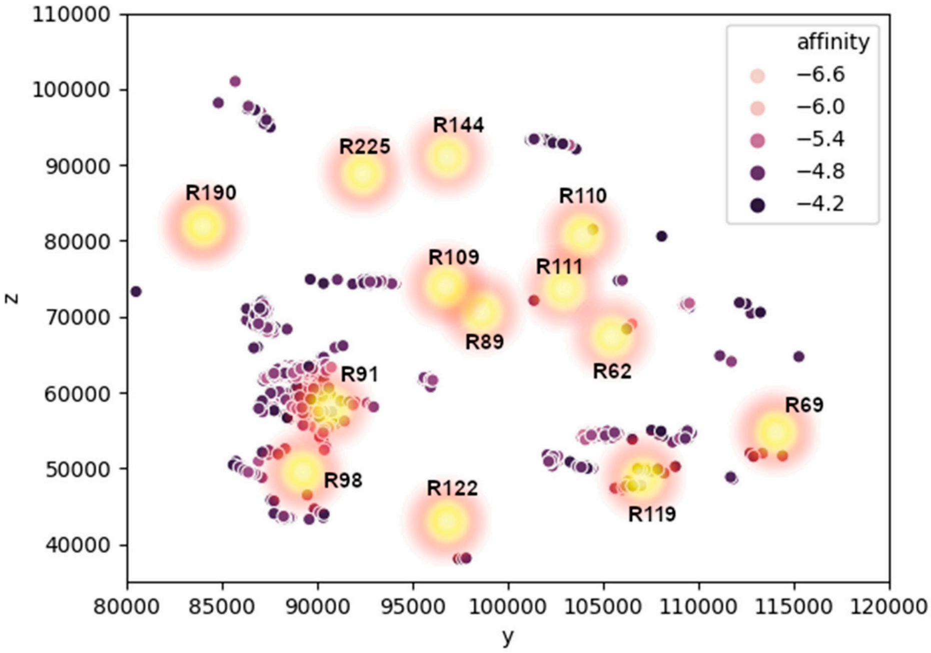

B

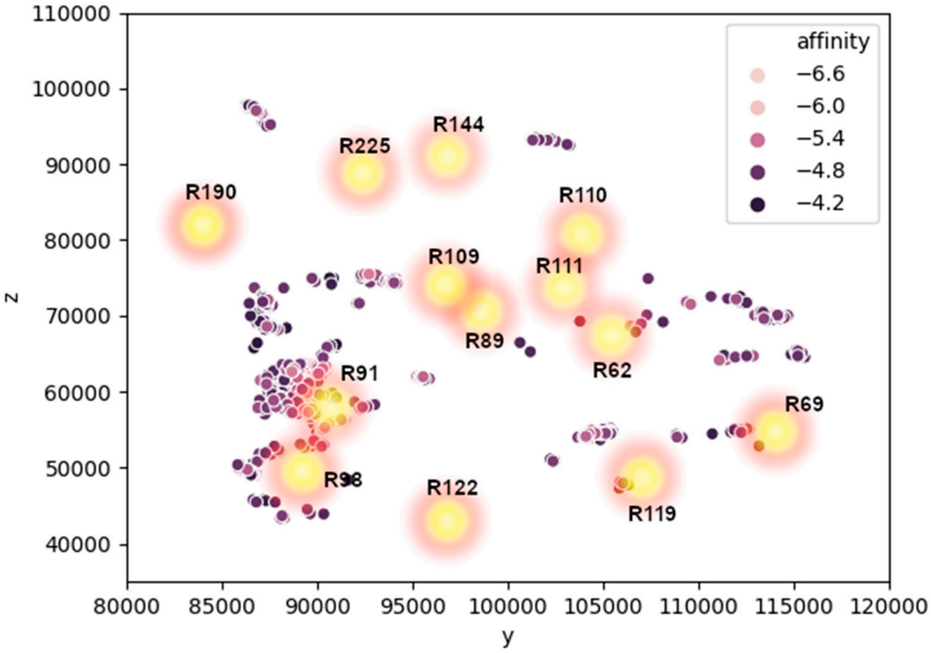

Supplementary Figure S6. Plot of spatial coordinates of sialic acid molecules and CD33 arginines. Bidimensional plot of areas around the atomic YZ coordinates of C_ζ of arginine residues (yellow and red circle) in the two-domain CD33 structures (PDB ID 5IHB). Overlay of carbon 1 YZ coordinates of the sialic acid structures binding the CD33 receptor form with the arginine (**A**) or glycine (**B**) residue in position 69.

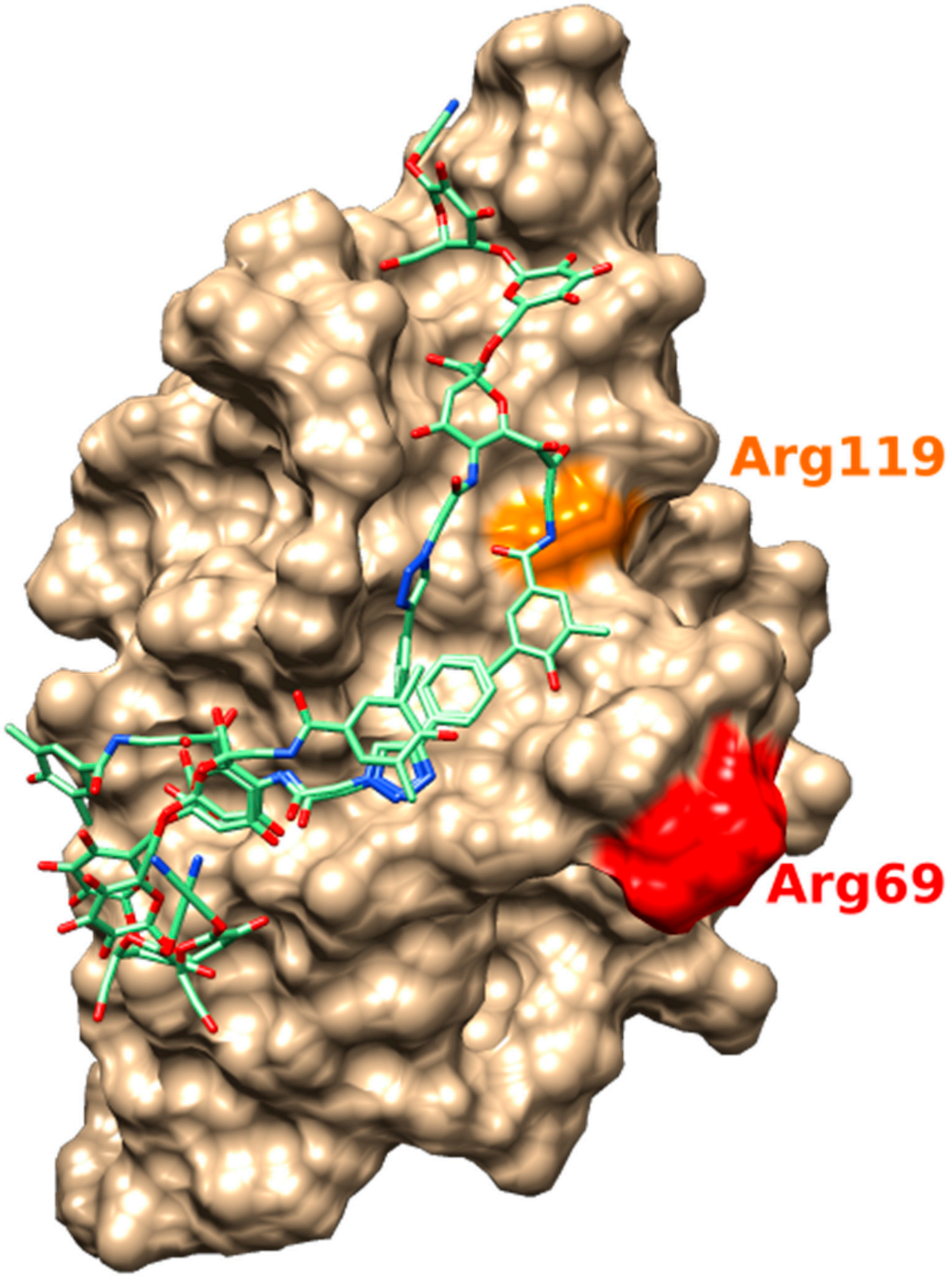

Supplementary Figure S7. Structural representation of CD33 N-terminal domain binding a subtype-selective sialic acid mimetic. Surface representation of the CD33 monomer (PDB ID 6D49), reporting the putative binding site to the binding with FVP.

Supplementary Table S1. List of the human CD33 structures (Siglec-3) available in Protein Data Bank.

Protein ID	Chains Number [a]	Resolution (Å)	Residue in position 69	Ligands [b]	References
5IHB	4	2.24	Gly	NAG	10.2210/pdb5IHB/pdb
5J06	4	2.66	Gly	GAL, NAG, PG0, SIA	10.2210/pdb5J06/pdb
5J0B	4	2.48	Gly	GAL, NAG, PG0, SIA	10.2210/pdb5J0B/pdb
6D48	4	1.78	Arg [c]	--	10.2210/pdb6D48/pdb
6D49	2	1.80	Arg	FVP, GOL	10.2210/pdb6D49/pdb
6D4A	2	1.75	Arg	FVP, GOL	10.2210/pdb6D4A/pdb
7AW6	2	1.95	Arg	FVP	10.2210/pdb7AW6/pdb

[a] Referred to the asymmetric unit assembly.

[b] NAG, 2-acetamido-2-deoxy-beta-D-glucopyranose; GAL, N-acetyl-alpha-neuraminic acid-(2-3)-beta-D-galactopyranose; PG0, 2-(2-methoxyethoxy)ethanol; SIA, N-acetyl-alpha-neuraminic acid; FVP, 2-aminoethyl 5-{[(4-cyclohexyl-1H-1,2,3-triazol-1-yl)acetyl]amino}-3,5,9-trideoxy-9-[(4-hydroxy-3,5-dimethylbenzene-1-carbonyl)amino]-D-glycero-alpha-D-galacto-non-2-ulopyranonosyl-(2->6)-beta-D-galactopyranosyl-(1->4)-beta-D-glucopyranoside; GOL, glycerol.

[c] For a deletion of 13 residues on the N-terminal of 6D48 structure, the Arg residue is numbered 57 in the aa sequence but overlaps to position 69 in the structure.
